# Evaluating personal care product use by Environmental Working Group hazard scores in relation to consumers’ sociodemographic characteristics, purchasing behaviors, and product safety perceptions

**DOI:** 10.1038/s41370-025-00751-9

**Published:** 2025-02-21

**Authors:** Emily S. Barrett, Karolin Wadie, Kylie Getz, Patricia Greenberg, Taina Moore, Adana A. M. Llanos

**Affiliations:** 1 Department of Biostatistics and Epidemiology, Rutgers School of Public Health, Piscataway, NJ USA; 2 Environmental and Occupational Health Sciences Institute, Rutgers University, Piscataway, NJ USA; 3 Department of Toxicology, Ernest Mario School of Pharmacy, Rutgers University, Piscataway, NJ USA; 4 Department of Epidemiology, Mailman School of Public Health, Columbia University Irving Medical Center, New York, NY USA; 5 Herbert Irving Comprehensive Cancer Center, Columbia University Irving Medical Center, New York, NY USA

**Keywords:** Personal care products, endocrine disrupting chemicals, environmental health disparities, commercial determinants of health, Environmental Working Group

## Abstract

**Background:**

Personal care products (PCPs) are a source of environmental chemical exposures. Little research has examined the specific PCPs people use, the environmental hazards posed by those PCPs, and factors informing PCP selection.

**Objective:**

To examine chemical hazards of the specific products used in relation to sociodemographic factors, purchasing behaviors, and perceptions about PCP safety.

**Methods:**

In a cross-sectional, university-based sample (NJ, USA, *N* = 593), participants reported on sociodemographics, PCP purchasing behaviors and perceptions, and PCP use in the last 24–48 h (including brand and product name). Those PCPs were linked to product hazard scores (1=least hazardous, 10=most hazardous) in the Environmental Working Group’s Skin Deep® database. For each participant, we calculated average hazard scores across all PCPs used and by category (e.g., haircare, skincare) and evaluated use of PCPs with high hazard scores (7–10). We fitted adjusted regression models examining associations of sociodemographic factors and participants’ perceptions and purchasing behaviors with product hazard scores.

**Results:**

Of 9349 unique PCPs used by participants, 68% matched to Skin Deep®. Average hazard scores varied by participant characteristics (e.g., age) for perfumes/colognes, beauty, and skin care products. The relative risk (RR) of recent use of a hair product with a high hazard score was twice as high in non-Hispanic Black women compared to non-Hispanic White women (RR:1.99; 95%CI:1.37, 2.89). Frequent use of healthy product apps (β = −0.49, 95%CI:−0.77, −0.21), reading product ingredient labels (β = −0.26; 95%CI:−0.82, −0.30), and seeking eco-friendly products (β = −0.17; 95%CI:−0.36, −0.01) were associated with use of skin care products with lower hazard scores. Results for hair and beauty products were similar. Concerns about PCP health impacts and regulation were associated with using products with lower hazard scores.

**Impact Statement:**

Personal care products (PCPs) can contain numerous endocrine disrupting and carcinogenic chemicals. In a U.S. university-based sample, we linked the PCPs used by participants in the last 24–48 h to hazard scores in the Skin Deep® database. Average hazard scores of the PCPs used by participants varied by sociodemographic factors. Participant behaviors (e.g., use of healthy product apps) and perceptions of PCP safety and regulation were associated with the average hazard scores of the PCPs they used. Our findings suggest that education and tools to inform PCP choice may help consumers choose safer products and potentially, reduce chemical exposures.

## Introduction

The average consumer in the United States (U.S.) is exposed to more than one hundred chemicals each day through the use of personal care products (PCPs) such as hair care products, soaps and cleansers, lotions, perfumes, make-up, and cosmetics [1]. Many of the chemicals in PCPs, including phthalates, parabens, and per- and polyfluoroalkyl substances (PFAS), are known or suspected endocrine disruptors or carcinogens [2–4], and increasingly, use of certain PCPs has been linked with adverse health outcomes including reproductive cancers [5–9], uterine fibroids [10], and preterm birth [11]. Chemical analyses of specific PCPs, furthermore, have demonstrated hormone activity and hormonally active ingredients in hair products widely used by Black women [12, 13]. However, despite growing recognition of the potential hazards associated with PCP use, in the U.S., there is limited regulation of this multi-billion-dollar industry, leaving consumers vulnerable to the potential risks posed by these chemicals [14–16].

Previous research has demonstrated disparities in PCP use by sociodemographic characteristics. For instance, in a recent survey of U.S. adults, females used 16 products daily on average versus nine products daily among males [17]. Other work has demonstrated significant differences in patterns of PCP use among women by race and ethnicity, with marginalized groups often using more products [18, 19], which may reflect pressures to conform to Eurocentric beauty norms [20]. In a study of U.S. pregnant people, those without a college degree or who did not have private health insurance used more types of PCPs in early pregnancy and were more likely to use perfume across pregnancy than those with college degrees or private insurance [21]. Similarly, among Canadian pregnant women, higher income was associated with less use of PCPs across multiple time points in gestation through the postpartum [22]. Studies of younger, college age women have also reported more extensive PCP use than studies of older women [23–25]. To date, most studies in this field have focused on the overall number of products used or the frequency of use of particular types of products (e.g., multiple times a day, once a week). The majority of studies have not considered the exact brands and formulations used. Collecting more granular data on how sociodemographic factors may contribute to differences in the exact PCPs used by consumers is an important next step given that even within categories of products (e.g., hair care products), ingredients lists can vary dramatically with some products containing far more potentially carcinogenic and endocrine-disrupting chemicals than others [26].

Perceptions about the safety of PCPs may also impact the products people choose and by extension, their exposure to the chemicals contained therein. For instance, in a small study of 72 college students, most participants (87%) expressed concerns about toxic chemicals in the home and PCPs, and 73% believed that the products they use could impact their health [27]. In our prior study of university-affiliated adults, 45% reported avoiding certain PCPs due to concerns about allergic reactions or other health impacts and there was moderate agreement that PCP use may impact health, with some variation noted by gender, race, and ethnicity [17]. In an analysis focused on hair product use in particular, we additionally found that participants with stronger concerns about PCP safety were more likely to practice “safer” PCP behaviors, including frequently using healthy product smartphone apps, reading ingredient labels, and looking for natural, non-toxic, or eco-friendly labels when choosing hair care products [28]. In a study of women from California, Black, White, and multiracial women were more likely to report trying to avoid certain ingredients in PCPs as compared to Latina and Vietnamese women [29]. It remains unknown, however, whether concerns about potential health impacts associated with PCPs translate into using “safer” products.

To fill these gaps, we leveraged data from a survey of U.S. adults regarding the specific PCPs they use, their PCP purchasing habits, and their perceptions about the safety of PCPs. We linked participants’ PCP use data with hazard scores for those specific PCPs as determined by the Environmental Working Group’s Skin Deep® database [26]. Using these linked data we examined variation in the hazard scores of the PCPs used in the last 24–48 h in relation to: (1) participants’ sociodemographic characteristics; (2) their perceptions about PCP safety and regulation; and (3) their purchasing behaviors (e.g., use of healthy product smartphone apps, reading ingredient lists, seeking products with labels indicating the product is made with natural, non-toxic, or eco-friendly ingredients).

## Methods

### Study overview

From September–October 2019, adults affiliated with Rutgers University were recruited into a study on their use of PCPs, their perceptions and attitudes regarding PCPs, and their PCP purchasing behaviors [17]. Recruitment occurred through listservs and email blasts sent to the Rutgers community. Eligibility criteria included having a Rutgers e-mail address and being age 18 or older. Participation consisted of a single 30–40 min online questionnaire administered through Qualtrics. To facilitate accurate reporting, participants were asked to complete the questionnaire at home while they had access to the PCPs they had used within the last 24–48 h. The Institutional Review Boards at Rutgers University (Pro2019000563) and Columbia University (AAAU4073) approved this study, and all participants signed electronic informed consent prior to completing the study questionnaire.

### PCP Questionnaire

Participants completed items on sociodemographic characteristics followed by a series of questions on PCP use. Participants were asked to provide detailed information on the specific products used within the last 24–48 h by category: hair care (e.g., shampoo, conditioner, styling gel), beauty (e.g., foundation, mascara, lipstick), skin care (e.g., moisturizers and cleansers for the face or body, body scrubs), oral care (e.g., toothpaste, dental floss, mouthwash), and perfumes/colognes. Participants were additionally queried about use of intimate care and menstrual products and were asked to provide data on other PCPs (e.g., petroleum jelly) that did not clearly fit into the other previously designated categories. For each product, participants reported the brand and specific product name as well as the color or scent, when applicable.

Participants responded to a series of seven questions on their perceptions about the safety and regulation of PCPs (Supplemental Table 1). For each item, response choices included strongly disagree, disagree, neither disagree nor agree, agree, or strongly agree. We additionally asked a series of questions on participant behaviors related to PCP purchasing, specifically with regards to practices that may help them choose safer products (Supplemental Table 1). Participants responded to these three items specifically in relation to skin, hair, and beauty product-related behaviors. For each item, response choices included never, rarely, sometimes, usually, or always.

### Product hazard scores

After data cleaning by trained study staff, the specific PCPs used in the last 24–48 h were matched to hazard scores in the Environmental Working Group (EWG)’s Skin Deep® database (date: 7/14/22) [26], a free, publicly available online resource made for consumers to assess the safety of specific PCPs. There are no fees for products to be included in Skin Deep® and products can be added by brands, retailers, or directly by consumers/users. The Skin Deep® database currently includes over 100,000 products and 3500 brands, and is regularly updated to add new products or delete discontinued ones.

The process of generating a hazard score for a given PCP is described in detail on the Skin Deep® website (https://www.ewg.org/skindeep/learn_more/about/) [26]. Briefly, EWG has developed an integrated database on health hazards and regulatory standards for over 1500 chemicals included in PCPs. The database aggregates information from over 50 high quality sources including the U.S. Environmental Protection Agency (E.P.A.), the International Agency for Research on Cancer (I.A.R.C), the U.S. Food and Drug Administration (F.D.A.), and the National Toxicology Program (N.T.P.). For each PCP included in the database, staff scientists first link each of the product’s ingredients to their integrated database, generally based on Chemical Abstract Service (C.A.S.) number. Based on the evidence, each ingredient is assigned a score of 0–100 (with 100 being most hazardous) in each of 17 hazard categories (cancer, developmental and reproductive toxicity, allergies and immunotoxicity, use restrictions, endocrine disruption, neurotoxicity, organ system toxicity, biochemical and cellular level changes, persistence and bioaccumulation, ecotoxicity, irritation, occupational hazards, enhanced skin absorption, and contamination concerns). For example, a known carcinogen would receive a score of 100 for the cancer hazard category. For each ingredient, points are assigned to each hazard category, weighted, and summed to create a raw score, which is then additionally weighted based on absorption potential (e.g., use of penetration enhancers and nanoparticles). Details on weighting factors are described more extensively on the Skin Deep® website [26].

Next, looking across all of the ingredients within a product, for each individual category (e.g., immunotoxicity), the points for the highest scoring ingredient (i.e., most hazardous) are added to the average scores for the other ingredients, and that sum is again weighted for absorption potential to create an overall product score. For comparability, all product and ingredient scores are rescaled from 1–10 with the 5% most hazardous ingredients and products receiving a score of 10, and so forth (scaling uniformly down to 1, indicating least hazardous).

For the current analysis, using the product names provided by participants and cleaned by study staff, an EWG staff scientist linked those PCPs to the EWG Skin Deep® database’s hazard scores based on product name. For products that did not match during the initial linkage, the study team manually evaluated whether they matched products listed in Skin Deep® but had, for instance, failed to link due to slightly different wording in the product name. Products that did not match to the EWG Skin Deep® database as of July 14, 2022 through either the automated or manual process were classified as having unknown hazard scores. Coverage of intimate care and menstrual products in Skin Deep® was very limited at the time of analysis and therefore that category of products was excluded from further analysis.

### Statistical analysis

The analytic sample included participants who provided demographic information as well as data on their recent PCP use. We first examined descriptive statistics (e.g., mean, standard deviation, min, max, n, frequency, missingness) for all variables of interest. Sociodemographic characteristics considered included age (categorized as 18–39, 40–59, and 60+ years old), gender (male, female), education (categorized as less than bachelors, bachelors, and more than bachelors), income (categorized as <$50,000, $50,000–74,999, $75,000–99,999, ≥$100,000), race and ethnicity (categorized as Asian/Pacific Islander, Hispanic, non-Hispanic Black, non-Hispanic White, multiracial or other groups), marital status (married versus unmarried), and role at the university (categorized as student, faculty, and staff). We then evaluated average hazard scores (across all products reported as well as by product category) in relation to the sociodemographic factors using Kruskal-Wallis tests. To assess proportion of products that had an exact match in the Skin Deep® database (overall and by product category) by race and ethnicity, we used chi-squared tests.

We fitted a series of multivariable linear regression models using average PCP hazard scores as the outcome. First, we examined associations between sociodemographic characteristics (age, race and ethnicity, marital status, income, and gender) and average hazard scores for PCPs used in the last 24–48 h, overall and by product category. Next, we evaluated average hazard scores (across all products) in relation to perceptions concerning PCP safety, adjusting for the sociodemographic factors above. For each of the seven perceptions around PCP safety and regulation, we fitted a linear regression model with the outcome average total hazard score (for all products used). For each model, “neither agree nor disagree” was designated as the reference group. Lastly, we fitted linear regression models to examine three environmental health-related purchasing behaviors (the frequency of using healthy product apps/websites, reading product labels for ingredients, seeking products with natural/non-toxic/eco-friendly labels) in relation to the average Skin Deep® hazard scores for hair care, skincare, and beauty products used by those participants. Models were again adjusted for the same set of sociodemographic characteristics specified above. Secondarily, given the potential health risks that may be associated with use of PCPs with the highest hazard scores (defined by EWG and here as a score of 7 or above), we refitted all models to examine the relative risk of using one or more products (overall and within categories) with a hazard score of 7 or above, again adjusting for the same set of covariates. All analyses were conducted in R Version 4.2.1.

## Results

### Descriptive statistics

In total, 593 participants provided complete PCP data for use in the current analysis. Of those, 510 (86%) identified as females and 83 (14%) identified as males (Table 1). Over half (55.6%) of the sample identified as non-Hispanic White, with smaller percentages identifying as Asian/Pacific Islander (19.3%), Hispanic (10.3%), non-Hispanic Black (9.3%), or multiracial or other groups (5.4%). The majority of participants (60.2%) were aged 18–39 years and not married (60.5%). Staff members comprised 41.7% of the sample with faculty (15.5%), graduate students (23.6%), and undergraduates (14.8%) accounting for smaller portions. Highest level of education was heterogeneous, reflecting these diverse roles, and overall, just over half of the sample (53.8%) reported an annual family income of over $100,000.

**Table 1:** Descriptive statistics of analytic sample overall and by race/ethnicity (*N* = 593).

Characteristics	Overall, *N* = 593	Asian/Pacific Islander, *n* = 115 (19.3%)	Hispanic, *n* = 61 (10.3%)	Non-Hispanic Black, *n* = 55 (9.3%)	Non-Hispanic White, *n* = 330 (55.6%)	Multiracial or other groups^a^, *n* = 32 (5.4%)
Age Categories (years)						
18–39	357 (60.2%)	96 (83.5%)	46 (75.4%)	32 (58.2%)	160 (48.5%)	23 (71.9%)
40–59	155 (26.1%)	16 (13.9%)	13 (21.3%)	21 (38.2%)	99 (30.0%)	6 (18.8%)
60+	80 (13.5%)	< 5	2 (3.3%)	< 5	71 (21.5%)	< 5
Missing	1 (0.2%)	0 (0%)	0 (0%)	0 (0%)	0 (0%)	1 (1.8%)
Gender						
Female	510 (86.0%)	99 (86.1%)	53 (86.9%)	53 (96.4%)	278 (84.2%)	27 (84.4%)
Male	83 (14.0%)	16 (13.9%)	8 (13.1%)	2 (3.6%)	52 (15.8%)	5 (15.6%)
Hispanic ethnicity (yes)	61 (10.3%)	0 (0%)	61 (100%)	0 (0%)	0 (0%)	0 (0%)
US-born (yes)	491 (82.8%)	69 (60.0%)	41 (67.2%)	47 (85.5%)	311 (94.3%)	23 (71.9%)
Marital status						
Married	234 (39.5%)	29 (25.2%)	15 (24.6%)	14 (25.5%)	167 (50.6%)	9 (28.1%)
Non-Married^b^	359 (60.5%)	86 (74.8%)	46 (75.4%)	41 (74.5%)	163 (49.4%)	23 (71.9%)
Education						
Less than bachelors	154 (26.0%)	46 (40.0%)	22 (36.1%)	20 (36.4%)	59 (17.9%)	7 (21.9%)
Bachelors	186 (31.4%)	35 (30.4%)	19 (31.1%)	17 (30.9%)	100 (30.3%)	15 (46.9%)
More than bachelors	251 (42.3%)	34 (29.6%)	20 (32.8%)	18 (32.7%)	170 (51.1%)	9 (28.1%)
Missing/unknown	2 (0.3%)	0 (0%)	0 (0%)	0 (0%)	1 (0.3%)	1 (3.1%)
Household income						
<$50,000	92 (15.5%)	27 (23.5%)	12 (19.7%)	17 (30.9%)	30 (9.1%)	6 (18.8%)
$50,000–$74,999	84 (14.2%)	11 (9.6%)	15 (24.6%)	10 (18.2%)	40 (12.1%)	8 (25%)
$75,000–$99,999	90 (15.2%)	8 (7.0%)	13 (21.3%)	8 (14.5%)	56 (17.0%)	5 (15.6%)
≥$100,000	319 (53.8%)	68 (59.1%)	21 (34.4%)	19 (34.5%)	198 (60.0%)	13 (40.6%)
Missing	8 (1.3%)	1 (0.9%)	0 (0%)	1 (1.8%)	6 (1.8%)	0 (0%)
Role at university						
Undergraduate student	88 (14.8%)	40 (34.8%)	12 (19.7%)	9 (16.4%)	20 (6.1%)	7 (21.9%)
Graduate student	140 (23.6%)	35 (30.4%)	10 (16.4%)	14 (25.5%)	71 (21.5%)	10 (31.3%)
Faculty	92 (15.5%)	7 (6.1%)	< 5	5 (9.1%)	75 (22.7%)	< 5
Staff	247 (41.7%)	30 (26.1%)	32 (52.5%)	27 (49.1%)	145 (43.9%)	13 (40.6%)
Other/unknown	26 (4.4%)	3 (2.6%)	4 (6.6%)	0 (0%)	19 (5.8%)	< 5

^a^Multiracial or other groups category included those reporting more than one race, American Indian or Alaska Native, and prefer not to answer or no response.

^b^Non-married category included those who reported being single/never married, widowed, separated, or divorced.

### PCP hazard scores by sociodemographic factors

Participants reported using an average of 14.5 ± 7.6 PCPs in the prior 24–48 h (not shown). Of the 9349 products used by the participants in the analytic set in the prior 24–48 h, 67.9% (*n* = 6334) had an exact match in the Skin Deep® database as of July 14, 2022 (Table 2). Exact matches were highest among hair products (76.7%) and skincare products (75.4%) and lowest for perfumes and colognes (60.8%) as well as “other” PCPs (i.e., products that did not fall into any other PCP category; 53.5%). For the most part, the percentage of PCPs with an exact match in Skin Deep® did not differ based on the race and ethnicity of the participants who reported their use (Table 2). However, hair products used by non-Hispanic Black participants were less likely to be found in the database compared to hair care products used by other racial and ethnic groups (68.7% vs 74.5–81.7%, *p* = 0.02). Differences in exact matches for “other PCPs” by race and ethnicity were also observed however overall, the number of products reported in that catch-all category was small (*n* = 69 products total).

**Table 2:** Number and percentage of personal care products (PCPs) used by participants in the last 24–48 h that were found in the EWG Skin Deep Database as of July 14, 2022, overall and by product category as well as by participant race/ethnicity.

PCPs used that linked to products in the Skin Deep database	Overall unique products used, *N* = 9349	Products used by participant self-reported race and ethnicity, *n* (%)					
		Asian/Pacific Islander, *n* = 1710	Hispanic, *n* = 555	Non-Hispanic Black, *n* = 1073	Non-Hispanic White, *n* = 5102	Multiracial or other^a^, *n* = 881	*P*^b^
PCPs, overall	6334 (68.0%)	1138 (66.5%)	646 (67.9%)	712 (66.4%)	3504 (68.7%)	334 (69%)	0.36
Hair products	1314 (76.7%)	219 (77.9%)	138 (76.2%)	145 (68.7%)	740 (77.5%)	72 (84.7%)	0.02
Beauty products^c^	1414 (65.5%)	240 (62.5%)	137 (68.2%)	143 (67.1%)	828 (65.3%)	66 (70.2%)	0.50
Skincare products^d^	2315 (75.4%)	428 (74.4%)	233 (73.7%)	264 (76.3%)	1264 (76.2%)	126 (73.7%)	0.77
Perfumes and colognes	240 (60.8%)	48 (60.8%)	26 (57.8%)	37 (63.8%)	113 (60.1%)	16 (64%)	0.97
Oral care products	976 (83.8%)	194 (88.2%)	106 (90.6%)	105 (82.0%)	522 (81.6%)	49 (81.7%)	0.04
Intimate care products^e^	7 (1.0%)	< 5	2 (2.4%)	< 5	< 5	< 5	0.32
Other PCPs	68 (53.1%)	7 (38.9%)	4 (57.1%)	16 (84.2%)	36 (46.8%)	5 (71.4%)	0.03

^a^Multiracial or other groups category included those reporting more than one race, American Indian or Alaska Native, and prefer not to answer or no response

^b^*P*-values based on chi-squared tests comparing percentage of products found in Skin Deep across race/ethnicities.

^c^Beauty products included face makeup, eye makeup, lip products, and nail products.

^d^Skincare products included rinse-off and leave-on facial skin care products, rinse-off body cleansing products, skin moisturizing products, at-home hair removal products, antiperspirants and/or deodorants, and sunscreen, sun tanning, after sun products and/or sunless tanning products.

^e^Intimate care products include intimate wash and wipe products.

For the PCPs participants used that matched the Skin Deep® database, the distribution of hazard scores is shown in Supplemental Table 2; of note, perfumes and colognes tended to have higher hazard scores, while oral care products tended to have low to moderate hazard scores. We considered participants’ sociodemographic factors in relation to continuous hazard scores of the products they reported using (overall and by product category), observing some differences (Table 3). Participants aged 40-59 years tended to use beauty products with higher hazard scores (mean ± SD: 4.28 ± 1.23) than the younger (3.91 ± 1.26) and older (4.13 ± 1.02) groups. By contrast, hazard scores were higher for perfumes and colognes used by the youngest participants (7.22 ± 1.95) compared to the participants age 40–59 (6.78 ± 2.15) and 60+ (5.92 ± 2.59). Females tended to use hair products with higher hazard scores than males (5.02 ± 1.11 vs. 4.71 ± 1.28, respectively), while non-married people tended to use skincare products with higher hazard scores than married people (4.43 ± 0.92 vs. 4.25 ± 0.88). No significant differences in continuous PCP hazard scores were observed in relation to the other sociodemographic characteristics studied including race and ethnicity and education.

**Table 3:** Average EWG Skin Deep database hazard scores of personal care products (PCPs) used in the last 24–48 h (overall and by product category) in relation to participant sociodemographic characteristics^a^.

	PCPs, all	Hair products	Beauty products	Skincare products	Perfumes and colognes	Oral care products	Other PCPs
**Average Skin Deep hazard scores by participant sociodemographic characteristics**	Mean±SD	Mean±SD	Mean±SD	Mean±SD	Mean±SD	Mean±SD	Mean±SD
Age (years)							
18–39	4.34 (0.69)	4.91 (1.10)	3.91 (1.26)	4.36 (0.92)	7.22 (1.95)	3.44 (0.723)	3.87 (1.82)
40–59	4.45 (0.63)	5.10 (1.26)	4.28 (1.23)	4.31 (0.88)	6.78 (2.15)	3.37 (0.650)	3.68 (1.72)
≥60	4.35 (0.59)	5.08 (0.94)	4.13 (1.02)	4.43 (0.86)	5.92 (2.59)	3.38 (0.714)	3.67 (1.37)
*p*-value	0.26	*0.08*	**0.03**	0.26	**0.04**	0.50	0.66
Education							
Less than bachelors	4.46 (0.61)	4.96 (0.99)	4.08 (1.24)	4.50 (0.86)	6.91 (2.22)	3.44 (0.64)	3.95 (1.74)
Bachelors	4.34 (0.63)	5.06 (1.12)	3.88 (1.29)	4.31 (0.96)	6.95 (1.79)	3.43 (0.72)	3.85 (1.93)
More than bachelors	4.35 (0.71)	4.94 (1.22)	4.15 (1.16)	4.33 (0.89)	7.00 (2.33)	3.40 (0.71)	3.63 (1.57)
*p*-value	0.15	0.80	0.36	*0.09*	0.88	0.21	0.75
Race and ethnicity							
AAPI	4.31 (0.67)	4.84 (1.30)	4.07 (1.19)	4.30 (0.89)	7.15 (1.90)	3.41 (0.64)	4.58 (1.69)
Hispanic	4.30 (0.68)	4.68 (0.97)	3.97 (1.49)	4.35 (0.94)	6.47 (2.35)	3.38 (0.67)	2.50 (3.00)
NHB	4.54 (0.58)	5.23 (1.28)	3.78 (1.23)	4.40 (0.94)	7.23 (1.52)	3.50 (0.76)	4.11 (2.03)
NHW	4.36 (0.68)	5.02 (1.03)	4.07 (1.18)	4.35 (0.91)	6.87 (2.31)	3.42 (0.73)	3.65 (1.33)
Multiracial or other^b^	4.49 (0.52	5.27 (1.28)	4.28 (1.46)	4.58 (0.88)	7.30 (1.87)	3.28 (0.61)	4.11 (1.94)
*p*-value	0.15	*0.04*	0.54	0.57	0.90	0.80	0.38
Gender							
Female	4.38 (0.65)	5.02 (1.11)	4.05 (1.19)	4.34 (0.89)	7.00 (2.08)	3.41 (0.68)	3.88 (1.70)
Male	4.29 (0.71)	4.71 (1.24)	3.82 (1.92)	4.49 (1.02)	6.59 (2.37)	3.47 (0.81)	2.75 (1.50)
*p*-value	0.33	**0.01**	0.63	0.12	0.52	0.75	0.17
Natality							
US-born (yes)	4.37 (0.67)	5.04 (1.09)	4.04 (1.20)	4.34 (0.90)	6.93 (2.18)	3.41 (0.709)	3.78 (1.64)
US-born(no)	4.34 (0.61)	4.71 (1.28)	4.03 (1.40)	4.44 (0.95)	6.96 (1.81)	3.46 (0.682)	3.81 (2.17)
*p*-value	0.96	*0.05*	0.90	0.42	0.33	0.89	0.99
Marital status							
Married	4.32 (0.69)	5.03 (1.19)	4.16 (1.22)	4.25 (0.88)	6.69 (2.37)	3.37 (0.70)	3.38 (1.46)
Non-Married^c^	4.40 (0.64)	4.95 (1.09)	3.97 (1.24)	4.43 (0.92)	7.13 (1.91)	3.45 (0.70)	3.99 (1.80)
*p*-value	0.19	0.28	0.18	**0.03**	0.32	0.10	0.23
Household income ($)							
<$50,000	4.33 (0.64)	4.72 (1.29)	3.88 (1.33)	4.38 (1.07)	7.00 (2.25)	3.39 (0.80)	4.87 (2.50)
$50,000–$74,999	4.52 (0.60)	5.05 (0.88)	4.08 (1.35)	4.61 (0.80)	6.96 (1.88)	3.42 (0.77)	3.97 (1.80)
$75,000–$99,999	4.39 (0.82)	5.15 (1.15)	4.02 (1.12)	4.16 (1.00)	7.01 (2.11)	3.53 (0.61)	2.80 (1.30)
≥$100,000	4.34 (0.62)	4.99 (1.14)	4.09 (1.21)	4.35 (0.84)	6.95 (2.17)	3.38 (0.67)	3.71 (1.55)
*p*-value	0.15	0.35	0.78	*0.05*	0.87	0.37	0.18
Role at university							
Undergraduate	4.39 (0.63)	4.86 (1.06)	4.19 (1.35)	4.41 (0.92)	6.84 (1.90)	3.42 (0.64)	4.42 (2.01)
Graduate student	4.37 (0.73)	4.95 (1.13)	3.80 (1.22)	4.38 (0.97)	7.52 (1.67)	3.50 (0.68)	3.69 (1.68)
Faculty	4.27 (0.58)	4.92 (1.23)	4.07 (1.23)	4.40 (0.75)	6.73 (2.71)	3.44 (0.73)	3.22 (1.49)
Staff	4.40 (0.66)	5.06 (1.14)	4.14 (1.15)	4.32 (0.94)	6.79 (2.11)	3.37 (0.70)	3.87 (1.85)
Other/unknown	4.35 (0.61)	4.95 (0.90)	3.90 (1.74)	4.41 (0.80)	6.58 (3.23)	3.33 (0.89)	4.00 (0)
*p*-value	0.41	0.45	0.23	0.95	0.47	0.66	0.64

*AAPI* Asian American/Pacific Islander, *NHB* non-Hispanic Black, *NHW* non-Hispanic White.

^a^*P*-values are based on Kruskal-Wallis tests. Associations significant at *P* < 0.05 are bolded and those at *P* < 0.10 are italicized.

^b^Multiracial or other race category included those reporting more than one race, American Indian or Alaska Native, and prefer not to answer or no response.

^c^Non-married category included those who reported being single/never married, widowed, separated, or divorced.

When we examined product hazard scores categorized as low (0–2), medium (3–6), and high (7–10), we observed differences by race and ethnicity of the participants who reported their use (Supplemental Table 3). For example, overall, 15.0% of the PCPs used by non-Hispanic Black participants had a high hazard score as compared to 9.8–11.1% among PCPs used by participants in the other racial and ethnic groups. These disparities were particularly notable for the hair products, with 27.6% of the hair products used by Black participants rated as hazardous versus 6.6–12.2% among other racial and ethnic groups. Similar, but non-significant trends were observed for perfumes and colognes whereby 75.5% and 72.9% of the products used by Black and AAPI participants (respectively) had high hazard scores versus 60.0–64.7% among the products used by other groups. Some differences in categorized overall product hazard scores (low, medium, high) were also observed in relation to other sociodemographic features of their users including age, education, and gender, with lower use of high hazard PCPs among older participants ( ≥ 60 years) versus younger and males versus females (Supplemental Table 4).

In multivariable linear regression models mutually adjusted for a set of sociodemographic factors (gender, age, race and ethnicity, marital status, and income), compared to products used by the youngest participants (age 18–39), products used by participants aged 40-59 tended to have higher mean hazard scores (total products β = 0.14; 95% CI: 0.00, 0.28), with stronger differences observed for beauty products specifically (β = 0.39, 95%CI: 0.09, 0.68) (Table 4). However, average hazard scores of perfumes and colognes used by those aged ≥60 years (β = −1.31, 95%CI: −2.43, −0.19) and to a lesser extent, those in the 40-59 years group (β = −0.45, −1.20, 0.30) were lower than the hazard scores of perfumes and colognes used by the youngest age group (18–39 years). Males tended to use hair products with lower hazard scores than females (β = −0.31, 95%CI: −0.60, −0.01) and we additionally observed that being married was associated with use of products with a lower average hazard score compared to being unmarried (total products: β = −0.14, 95% CI: −0.27, 0.00), an association that was strongest in the context of skin care products (β = −0.23, 95%CI: −0.41, −0.03). Finally, some differences in the hazard scores of skin care products used were observed in relation to income, however the pattern was inconsistent across income levels. Of note, no differences in mean PCP hazard scores were observed in relation to the race and ethnicity of participants and no sociodemographic factors were significantly associated with mean hazard scores for oral care products.

**Table 4:** Multivariable linear regression models examining associations between sociodemographic characteristics and average hazard scores for personal care products (PCPs) used in the last 24−48 h, overall and by product category (*N* = 593)^a^.

	All products	Hair products	Beauty products	Skin care products	Perfumes/ colognes	Oral care products
	β (95% CI)					
**Gender**						
Female	REF	REF	REF	REF	REF	REF
Male	−0.06 (−0.21,0.10)	−**0.31(−0.60, −0.01)**	−0.26 (−0.78,0.27)	0.15 (−0. 08,0.37)	−0.34 (−1.43,0.74)	0.11 (−0.07,0.28)
**Age (years)**						
18−39	REF	REF	REF	REF	REF	REF
40−59	*0.14 (0.00,0.28)*	0.11 (−0.14,0.36)	**0.38 (0.08,0.68)**	0.03 (−0.17,0.23)	−0.45 (−1.20,0.30)	−0.09 (−0.24,0.07)
60+	0.06 (−0.11,0.24)	0.14 (−0.18,0.46)	0.18 (−0.22,0.58)	0.15 (−0.11,0.40)	−**1.31 (−2.43, −0.19)**	−0.06 (−0.25,0.14)
**Race and ethnicity**						
NHW	REF	REF	REF	REF	REF	REF
AAPI	−0.06 (−0.21,0.01)	−0.07 (−0.34,0.19)	0.12 (−0.21,0.44)	−0.11 (−0.32,0.10)	0.03 (−0.82,0.87)	−0.04 (−0.20,0.12)
Hispanic	−0.09 (−0.28,0.09)	−0.30 (−0.64,0.03)	−0.05 (−0.43,0.36)	−0.05 (−0.32,0.10)	−0.66 (−1.69,0.36)	−0.09 (−0.29,0.12)
NHB	0.15 (−0.04,0.35)	0.28 (−0.08,0.64)	−0.26 (−0.68,0.15)	0.03 (−0.25,0.30)	0.16 (−0.78,1.11)	0.06 (−0.15,0.27)
Multiracial/other^b^	0.09 (−0.04,0.35)	−0.02 (−0.14,0.72)	0.25 (−0.29,0.78)	0.13 (−0.21,0.47)	0.28 (−1.14,1.69)	−0.18 (−0.44,0.08)
**Marital status**						
Non-married^c^	REF	REF	REF	REF	REF	REF
Married	−**0.14 (−0.27, −0.00)**	0.004 (−0.24,0.24)	0.04 (−0.25,0.33)	−**0.23 (−0.41,−0.03)**	−0.18 (−0.90,0.54)	−0.06 (−0.21,0.09)
**Household income**						
<$50,000	−0.05 (−0.22,0.11)	−0.23 (−0.54,0.07)	−0.06 (−0.44,0.31)	−0.05 (−0.28,0.18)	−0.23 (−1.17,0.72)	−0.04 (−0.22,0.14)
$50,000 - $74,999	0.14 (−0.03, 0.31)	0.07 (−0.23,0.37)	0.05 (−0.32,0.41)	0.19 (−0.05,0.42)	-0.03 (−0.94,0.87)	0.02 (−0.16,0.21)
$75,000 - $99,999	0.02 (−0.14,0.18)	0.14 (−0.14,0.42)	−0.05 (−0.38,0.29)	−**0.24 (−0.46,−0.01)**	0.17 (−.73,1.07)	0.14 (−0.03,0.31)
$100,000+	REF	REF	REF	REF	REF	REF

*AAPI* Asian American/Pacific Islander, *NHB* non-Hispanic Black, *NHW* non-Hispanic White.

^a^Models are mutually adjusted for all sociodemographic factors in the table (e.g., gender, age, race/ethnicity, marital status, income). Associations significant at *p* < 0.05 are bolded and those with *p* < 0.10 are italicized.

^b^Multiracial or other race and ethnicity category included those reporting more than one race or multiracial (52 [7.9%]), American Indian or Alaska Native (2 [3.1%]), and unknown (those who provided no response to race and/or ethnicity or responded, ‘prefer not to answer’ (11 [16.9%]).

^c^Non-married category included those who reported being single/never married, widowed, separated, or divorced.

When we examined the risks of using at least one PCP with a high hazard score (7 or above), some racial and sociodemographic differences were evident (Supplemental Table 5). In multivariable logistic regression models, Non-Hispanic Black participants had a higher risk of using one or more products with a high hazard score (RR: 1.23, 95%CI: 1.03, 1.46), an association driven by hair product use (RR: 1.99; 95%CI: 1.37, 2.89). Similar to the models considering the continuous outcome, males consistently had lower odds of using PCPs with high hazard scores compared to females (RR: 0.65, 95%CI: 0.49, 0.86), a pattern that was consistent across all product subtypes (RRs: 0.51–0.83). We also observed differences by age, with older participants having higher risk of using hair products with a high hazard score (RR age 40–59: 1.55; 95%CI: 1.09, 2.19; RR age 60 + : 1.40; 95%CI: 0.86, 2.29) compared to the youngest group (ages 18–39 years). At the same time, older participants were less likely to use perfumes/colognes with high hazard scores (RR age 40–59: 0.87, 95%CI: 0.70, 1.08; RR age 60 + : 0.55; 95%CI: 0.32, 0.95). No notable associations with marital status or income were observed in this set of models.

### PCP hazard scores in relation to perceptions regarding PCP safety and regulation

We examined average hazard scores of all products used by participants in the last 24-48 h in relation to their self-reported perceptions about the PCP safety and regulation (Table 5). In adjusted models, participants who strongly agreed that “*The personal care products I use affect my health*”, used products with a lower average hazard score (β = −0.26, 95%CI: −0.43, −0.08) compared to those who neither agreed nor disagreed, and a similar trend was observed for those who strongly disagreed with the statement (β = −0.22, 95%CI: −0.46, 0.03). Compared to those who neither agreed nor disagreed, participants who disagreed with the statement “*Organic, natural, non-toxic, or eco-friendly personal care products have fewer toxic chemicals than regular products*”, tended to have higher average PCP hazard scores (β = 0.27, 95%CI: 0.04, 0.49), whereas average hazard scores were non-significantly lower among participants who agreed or strongly agreed. When presented with the statement “*There is no reason to worry about the health effects from chemicals that might be in personal care products*”, compared to those who neither agreed nor disagreed, those who strongly disagreed used products with significantly lower average hazard scores (β = −0.19, 95%CI: −0.38, −0.001), whereas a trend towards higher scores was observed among those who strongly agreed (β = 0.55, 95%CI: −0.22, 1.31). Participants who strongly agreed (β = −0.26, 95%CI: −0.48, −0.05) or agreed (β = −0.14, 95%CI: −0.26, −0.01) that “*Organic, natural, non-toxic, or eco-friendly personal care products are just as effective as regular products* used products with lower average hazard scores than participants who neither agreed nor disagreed. Additionally, participants who strongly disagreed that “*The Food and Drug Administration (FDA) and other government agencies do a good job of regulating personal care products to ensure they are safe for consumers*” tended to use products with lower average hazard scores (β = −0.22, 95%CI: −0.38, −0.05), whereas average product hazard scores were non-significantly higher among those who agreed (β = 0.16, 95%CI: 0.01, 0.32) or strongly agreed (β = 0.16, 95%CI: 0.16, 95%CI: −0.24, 0.56) with the statement compared to the reference group. When we examined the participant perceptions and attitudes described above in relation to risks of using at least one product with a high hazard score (7–10) in the last 24–48 h, fewer consistent associations were observed (Supplemental Table 6).

**Table 5:** Regression models examining average Skin Deep hazard scores of all personal care products (PCPs) used in relation to participants’ perceptions and attitudes about PCP safety (*n* = 581).

Perceptions	β (95%CI)^a^
The personal care products I use affect my health.	
Strongly disagree	*−0.22 (−0.46, 0.03)*
Disagree	0.10 (-0.07, 0.28)
Neither agree nor disagree	REF
Agree	*−*0.09 (*−*0.22,0.05)
Strongly agree	*−***0.26 (***−***0.43,** *−***0.08)**
Organic, natural, non-toxic, or eco-friendly personal care products have fewer toxic chemicals than regular products.	
Strongly disagree	0.14 (*−*0.25, 0.53)
Disagree	**0.27 (0.04, 0.49)**
Neither agree nor disagree	REF
Agree	*−*0.03 (*−*0.17, 0.10)
Strongly agree	*−*0.10 (*−*0.27, 0.08)
Consumers should be concerned about the health effects of personal care products.	
Strongly disagree	*−*0.21 (*−*0.88, 0.45)
Disagree	*−*0.08 (*−*0.49, 0.33)
Neither agree nor disagree	REF
Agree	0.001 (*−*0.17, 0.18)
Strongly agree	*−*0.11 (*−*0.29, 0.08)
There is no reason to worry about the health effects from chemicals that might be in personal care products.	
Strongly disagree	*−***0.19 (***−***0.38,** *−***0.001)**
Disagree	*−*0.01 (*−*0.19, 0.17)
Neither agree nor disagree	REF
Agree	*−*0.04 (*−*0.37, 0.29)
Strongly agree	0.55 (*−*0.22, 1.31)
Overall, the benefits of using personal care products outweigh any risks from exposure to toxic chemicals that might be in these products.	
Strongly disagree	*−0.17 (−0.33, 0.001)*
Disagree	0.02 (*−*0.10, 0.16)
Neither agree nor disagree	REF
Agree	0.03 (*−*0.14, 0.20)
Strongly agree	*−*0.18 (*−*0.72, 0.36)
Organic, natural, non-toxic, or eco-friendly personal care products are just as effective as regular products.	
Strongly disagree	*−*0.06 (*−*0.42, 0.30)
Disagree	*−*0.04 (*−*0.21, 0.12)
Neither agree nor disagree	REF
Agree	*−***0.14 (***−***0.26,** *−***0.01)**
Strongly agree	*−***0.26 (***−***0.48,** *−***0.05)**
The Food and Drug Administration (FDA) and other government agencies do a good job of regulating personal care products to ensure they are safe for consumers.	
Strongly disagree	*−***0.22 (***−***0.38,** *−***0.05)**
Disagree	0.04 (*−*0.09, 0.18)
Neither agree nor disagree	REF
Agree	**0.16 (0.01, 0.32)**
Strongly agree	0.16 (*−*0.24, 0.56)

^a^Models are adjusted for gender, age, race/ethnicity, marital status, and income. Associations significant at *p* < 0.05 are bolded and those with *p* < 0.10 are italicized.

### PCP hazard scores in relation to PCP purchasing behaviors

**Fig. 1:**
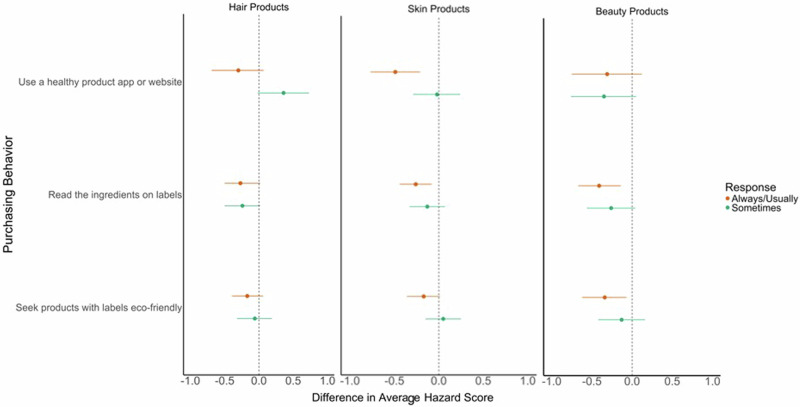
Adjusted linear regression models examining associations between personal care product (PCP) purchasing behaviors and average Skin-Deep hazard scores of PCPs used in the last 24–48 h, by product type^1,2^ (*n* = 593). ^1^Models adjusted for age, race/ethnicity, marital status, gender, and income. ^2^Reference group is respondents who answered that they rarely or never pursued the purchasing behaviors of interest.

Finally, we fitted linear regression models examining the relationship between the mean hazard scores of the hair, skin, and beauty products used in relation to participants’ self-reported PCP purchasing behaviors specific to those types of products. Compared to participants who reported rarely or never using healthy product apps or websites when purchasing products, those who always or usually used such apps or websites used hair (β = −0.30, 95%CI: −1.10, 0.68), skin (β = −0.49, 95%CI: −0.77, −0.21), and beauty (β = −0.31; 95%CI: −0.75, 0.12) products with lower average hazard scores (Fig. 1; Supplemental Table 7). Similarly, compared to those who reported rarely or never reading ingredients on PCP labels, those who always or usually read the ingredients used hair (β = −0.27, 95%CI: −0.49, −0.04), skin (β = −0.26, 95%CI: −0.82, −0.30), and beauty (β = −0.40; 95%CI: −0.67, −0.14) products with lower average hazard scores. Average PCP hazard scores were similarly lower for hair (β = −0.17, 95%CI: −0.39, 0.06), skin (β = −0.17, 95%CI: −0.36, −0.01), and beauty (β = −0.34, 95%CI: −0.62, −0.06) products used by those who reported always or usually seeking products with labels indicating natural, non-toxic, or eco-friendly ingredients compared to those who reported never or rarely seeking such products. When we examined the relative risks of using at least one product with a high hazard rating within the last 24–48 h, results were mostly attenuated (Supplemental Table 8).

## Discussion

In this study of PCP use among adults affiliated with a large U.S. university, we observed that for certain types of PCP, specifically hair products, beauty products, skincare products, and perfumes and colognes, the average hazard scores of products used differed by sociodemographic factors including age, gender, income, and marital status. Additionally, we observed higher risk of recent use of hair products with high hazard ratings among non-Hispanic Black women, middle aged participants, and females. Furthermore, we observed that participants’ perceptions around the health impacts of PCPs and the regulation of these products by government agencies were associated with the hazards scores of the products they used, with participants who expressed concern about the safety and regulation of PCPs generally using products with lower average hazard scores. Importantly, our results also suggested that behaviors such as use of healthy product apps and websites and reading ingredient labels were associated with use of products with lower hazard scores. By linking the specific products used by participants to data on the hazard profiles of those products, these results represent an important extension of the prior research which has more generally characterized PCP use by type or category of product.

Prior literature has documented sociodemographic differences in the number and type of PCPs used as well as the frequency of use. For example, in a prior analysis of this same university-based sample, we previously showed that Asian and Pacific Islander females used fewer hair products and more intimate care and menstrual products than non-Hispanic White females, while non-Hispanic Black females used more hair products, perfumes, oral care, and intimate care and menstrual products than non-Hispanic White females [17]. In a community-based study in California, Latina women tended to be heavier makeup users (in terms of product number and frequency) than other groups, while use of fragrances and certain types of hair products (including those linked to reproductive cancers and preterm birth) was most common among Black women [29]. Other research in women from New York City reported that African-American and African-Caribbean women were more likely to use a variety of hair products compared to White women, including products known to contain extracts of placenta (a hormonally active tissue) or other endocrine disruptors [30]. While the average hazard scores of the specific PCPs used by participants in the last 24 h did not differ by race and ethnicity, when we looked at the use of products with high hazard scores specifically, it emerged that non-Hispanic Black females were significantly more likely than non-Hispanic White females to have recently used a PCP with a high hazard score, a result driven by the use of hair products with high hazard scores. A number of prior studies have demonstrated persistent racial disparities in exposure to chemicals commonly found in PCPs (such as phthalates) and differences in PCP use have been proposed as a primary contributor to the unequal exposure burden observed among some race and ethnicity groups [30–33]. Our prior work in this cohort further showed that Black females used more haircare products and perfumes than other racial and ethnic groups [17]. The use of more hair products as well as hair products with higher hazard ratings among Black females may be an important contributor to health disparities and warrants further investigation including evaluation of specific patterns of use of products such as hair relaxers and oils as well as drawing distinctions between rinse-off and leave-on products which we were not able to do here.

We also observed that a disproportionately higher number of hair products used by diverse participants were not found in the Skin Deep® Database and thus their hazard profiles are unknown. This raises concerns about equitable public access to safety information, which is particularly important considering previous research linking hair products containing EDCs to breast cancer risk in African American women [34]. Of note, EWG also recognizes similar concerns and has prioritized the addition of products marketed to Black females in recent Skin Deep® updates with 1600 products being added in early 2024 alone (personal communication). Additionally, our findings highlight the need for further research in this area with more diverse and inclusive samples. Prior research focusing on males found significant correlations between PCP use and increased urinary concentrations of EDCs [35], as well as an increased risk of prostate cancer associated with hair dye use [9]. In our prior research in this sample, we observed that males used fewer products than females [17] and we add to those results here by showing that average hazard scores of products used by male respondents were similar to those used by females, suggesting potential risks to males as well. At the same time, for every product category we studied, males had a lower risk of having used a product with a high hazard score in the last 24–48 h compared to females, again showing that the greatest risks associated with PCP use may be to females. The existing literature and our current findings emphasize the importance of studies with diverse samples of both males and females to better understand the differences in PCPs used and their associated hazards. Further, inclusion of individuals belonging to sexual and gender minority populations including non-binary and transgender females, who are also frequent users of PCPs, must also be prioritized.

Some differences in PCP hazard scores were noted by participant age as well. Of particular note, the perfumes and fragrances used by younger participants have higher hazard scores than those used by the older groups. This is of concern given that of all product types, perfumes and fragrances tend to have the highest hazard scores due to the widespread inclusion of known endocrine disruptors like phthalates in fragrance formulations. These concerns are further heightened by recent media reports suggesting rising interest in and use of fragrances and other scented PCPs among even tweens and teenagers [36, 37]. At the same time, in our study, overall, average hazard PCP scores were highest among participants aged 40–59, a result that appeared to be mostly driven by use of beauty products. This may reflect conscious or unconscious societal pressures to adhere to beauty standards that favor youth leading to the use of products containing ingredients that include anti-aging or other “active” ingredients that may potentially have higher hazard scores. Because prior literature has largely focused on race and ethnicity-based disparities in PCP use or on limited age-bands (e.g., college students or reproductive-age women), little is known about how age influences product choice and use. While more work is needed to understand these results, it is important to note that like gestation, puberty, and pregnancy, the perimenopause is increasingly viewed as another life stage during which hormonal axes are in flux and may be more vulnerable to disruption by chemical exposures [38, 39].

Participants’ perceptions regarding the safety of PCPs and the effectiveness of government regulations were associated with the hazard scores of the products they used. Generally, participants who expressed greater concern about product safety or the effectiveness of regulations tended to use products with lower average hazard scores. In a prior analysis in this sample, we demonstrated that participants who agreed that PCPs could have harmful health impacts were more likely to use healthy product smart phone apps and websites or to read ingredient labels [28]. Collectively, these results suggest that educating consumers around potential hazardous exposures in PCPs and current U.S. policies around their regulation could help to inform consumer choices.

These observations raise important issues regarding environmental health literacy- namely how people conceptualize and contextualize environmental health information to shape their behaviors [40, 41]. Specific to PCPs, for example, some consumers may assume that the U.S. government closely regulates the products on the market, testing them for their safety, when in reality, there is very little safety testing beyond color additives [14, 42]. Choosing safer products therefore requires some environmental health literacy to navigate the vast PCP market. At present, research on environmental health literacy specific to PCPs is limited, although a recent study focused on phthalates, a common ingredient in PCPs, indicated race- and ethnicity-based disparities in knowledge around sources of exposure and health impacts [43]. In that study, participants who demonstrated greater knowledge of phthalates were also more likely to self-report avoiding PCPs containing phthalates. Together, results of that research and the current study indicate that improving environmental health literacy around chemicals in PCPs, particularly in minoritized communities, may shift PCP use behaviors and in so doing, be an effective approach to reducing known disparities in chemical exposures.

Additional research is needed to determine the extent to which use of “safer” products (i.e., those with lower hazard scores) translates to body burden of chemicals commonly found in PCPs, such as phthalates and phenols. In one study of teenage girls, replacing the usual PCPs they used for “cleaner” alternatives for 3 days resulted in a roughly 25–50% reduction in certain urinary phthalate and paraben metabolites [44]. Importantly, after that intervention, 71% of participants said they would subsequently buy products without phthalates, parabens, triclosan, and oxybenzone. Similar studies are needed to understand whether such reductions in body burden of EDCs can be replicated in real-life settings when consumers are empowered with knowledge and tools to independently choose safer products rather than being provided with pre-selected PCPs.

To that end, we observed differences in PCP hazard scores of products used by participants in relation to their self-reported purchasing behaviors. Participants who reported using healthy or “clean” product apps, regularly reading ingredient labels, and looking for labels indicating natural, non-toxic, or eco-friendly ingredients used PCPs with lower average hazard scores, suggesting that apps and labels may be effective in helping consumers to select safer products. At the same time, this is an imperfect solution as some prior work has noted that certain ingredients are often undisclosed on product labels (e.g., cyclosiloxanes) or are “hidden” under proprietary terms like “fragrance” [12, 45]. It also puts the burden on the consumer to research products and navigate complicated ingredient lists, which may be increasingly difficult with the recent rise in “clean washing”, or the false advertisement of products to appear safer to consumers [46, 47].

Many of the associations observed were small in magnitude, for instance differences of less than 1 in average hazard scores between those who use healthy product apps and websites versus those who do not. At present, the clinical relevance of these differences in terms of individual participants’ health is not clear, however, on a population level, even small differences may increase the burden of disease. In addition, the consistent daily use of PCPs over years or even decades is such that even minor differences in chemical exposures at one time point may add up to considerably higher cumulative lifetime exposure compared to those who use products with slightly lower hazard scores. Lastly, our observation that some demographic groups had higher risk of recent use of a PCP with high hazard ratings (e.g. Black women and hair products) suggests priorities for future work linking product use to health outcomes.

Structural barriers may also reduce access to safer products for some populations. For example, in a study of eight Boston neighborhoods, retail stores in lower income neighborhoods (particularly those in communities of color) carried a higher percentage of hair products that were rated as highly hazardous in the Skin Deep® database compared to retail stores in wealthier neighborhoods [48]. That study also noted that higher priced hair products were less likely to have average to high hazard scores (compared to lower priced products), however stores in lower SES neighborhoods were more likely to carry lower priced hair products. This suggests that in addition to potential knowledge barriers, accessing safer PCPs can be a challenge and ultimately, perpetuate disparities in EDC exposures. Notably, while we observed some racial and ethnic differences in the hazards of the particular PCPs used by participants, it is also possible that disparities are less pronounced among communities of higher average socioeconomic status (such as in an academic setting like the one our sample represents) who have access to internet retailers and reliable transportation to access multiple brick and mortar retailers. In a general population, even stronger racial and ethnic differences in PCP hazards might be observed.

We note multiple strengths of the current work. In contrast to most prior research in this area which queried number and types of products used, we collected comprehensive data on the specific products used including brand, formulation, and color. This granularity allowed us to link the specific products used to the Skin Deep® database, which provided a quantitative measure of product hazards. Additionally, our sample was more diverse in age and gender than prior studies, most of which have focused on reproductive age females. While that group is clearly one of interest given concerns about the reproductive and developmental toxicity of chemicals found in PCPs (e.g., phthalates, phenols), increasingly, evidence suggests that health risks associated with chemical exposures in PCPs may be relevant to all gender identities, with multiple critical windows of susceptibility across the lifespan. Additional work is needed to understand PCP use in males, older females (who tended to use some types of products with higher average hazard scores (e.g., beauty products)), and non-binary and transgender individuals. Finally, we included items on perceptions of harm associated with PCP use, an area that has received relatively little attention to date, but is important in terms of understanding consumer behaviors and informing future intervention strategies.

At the same time, there are several limitations of this research that should be considered. We did not collect details on the use of the PCPs such as the amount of product applied, how many times it was used during the prior 24–48 h, and whether the product was left on or rinsed off, all of which may impact chemical absorption and potential toxicological effects [49]. Leave on products, in particular, may have higher levels of certain phthalates and parabens [3]. Importantly, many products did not match to the Skin Deep® database resulting in missing data on hazard scores. Because products enter the database through reporting by brands, retailers, or consumers, smaller brands that are carried by fewer retailers and used by fewer consumers may be less likely to be added to the database. Among the products that commonly did not match to the Skin Deep® database were intimate care products (e.g., maxi pads, tampons, wipes, douches), oral care products (e.g. flosses, mouthwashes), direct to consumer brands (e.g., Avon, Mary Kay), and certain store brands (e.g. Bath and Body Works, generic brands like CVS Health and Up & Up). By necessity, we pursued a complete case approach and results could have been biased in either direction as a result. With the ongoing expansion of coverage of products in Skin Deep®, particularly those used by people of color, missingness may be less of an issue in future studies. This expansion is essential not just for future research, but to help all consumers make more informed decisions about their PCPs. In addition, PCP formulations change over time and we are unable to verify that the PCPs used by participants are the exact same formulations (e.g. same ingredient profile) as when those products were entered into Skin Deep®. While other databases exist to evaluate risks associated with certain PCPs, we opted to use data from Skin Deep® as it is particularly widely-used and well-known, and its hazard scores were developed based on 17 evidence-based criteria. We additionally note that our sample, based in an academic community, was relatively homogeneous and tended to be well educated with higher income, which may limit generalizability.

In summary, in this study of university-affiliated U.S. adults, we observed that the chemical hazards encountered through PCPs varied by consumers’ sociodemographic characteristics. We additionally observed that people’s perceptions around PCP safety and regulation were associated with the hazards of the products they used, suggesting the importance of education and environmental health literacy in shaping consumer choices. Finally, our results suggested that use of smartphone apps and websites promoting cleaner, safer beauty choices may be an effective way to help consumers choose PCPs that may pose fewer health hazards. These results suggest that individual-level behaviors may help to protect consumers from potentially harmful chemical exposures in PCPs in the absence of much-needed updates to U.S. regulations on the chemical ingredients found in PCPs.

## Supplementary information

Supplemental Material

## Data Availability

The data presented here are available from the corresponding author upon reasonable request (including IRB approval). They are not publically available due to privacy restrictions.
