# Supplementary material for: Evaluating personal care product use by Environmental Working Group hazard scores in relation to consumers’ sociodemographic characteristics, purchasing behaviors, and product safety perceptions
Source: J Expo Sci Environ Epidemiol. 2025 Feb 21;35(6):921–32. doi: 10.1038/s41370-025-00751-9 (PMC12583145; doi:10.1038/s41370-025-00751-9)
Supplement: Supplementary file 1 — Supplemental Material [file 41370_2025_751_MOESM1_ESM.docx]

**Supplemental Material**

| **Supplemental Table 1. Questionnaire items on personal care product (PCP)-related perceptions and purchasing behaviors** |
| --- |
| **Please indicate how much you agree or disagree with the following statements.**  **[choices: strongly disagree, disagree, neither disagree nor agree, agree, strongly agree]** |
| The personal care products I use affect my health |
| Organic, natural, non-toxic or eco-friendly personal care products have fewer toxic chemicals than regular products. |
| Consumers should be concerned about the health effects of personal care products. |
| There is no reason to worry about the health effects from chemicals that might be in personal care products |
| Overall, the benefits of using personal care products outweigh any risks from exposure to toxic chemicals that might be in these products |
| Organic, natural, non-toxic or eco-friendly personal care products are just as effective as regular products. |
| The Food and Drug Administration (FDA) and other government agencies do a good job of regulating personal care products to ensure they are safe for consumers. |
| **When buying [*product type*] products to use at home… ***  **[choices: never, rarely, sometimes, usually, always]** |
| …how often do you use a healthy product app or website (e.g. EWG, SkinDeep)? |
| …how often do you read the ingredients on the label? |
| …how often do you look for labels indicating the product is made with natural, non-toxic or eco-friendly ingredients? |

* This series of questions was repeated for skin care products, hair care products, and beauty products.

| **Supplemental Table 2. Distribution of hazard scores among the personal care products (PCPs) used by participants that linked to the Skin Deep database (n=6334).** | | | | | | | | | | | | |
| --- | --- | --- | --- | --- | --- | --- | --- | --- | --- | --- | --- | --- |
|  |  |  | **Skin Deep Hazard Score; n (%)** | | | | | | | | | |
| **PCPs used that linked to the Skin Deep database** | **Unique products used** | **0** | **1** | **2** | **3** | **4** | **5** | **6** | **7** | **8** | **9** | **10** |
| PCPs, overall | 6334 | 10 (0.2) | 258 (4.1) | 385 (6.1) | 1293 (20.4) | 1440 (22.7) | 1572 (24.8) | 698 (11.0) | 403 (6.4) | 147 (2.3) | 72 (1.1) | 56 (0.9) |
| Hair products | 1314 | 2 (0.2) | 51  (3.9) | 32 (2.4) | 45 (3.4) | 238 (18.1) | 498 (37.9) | 276 (21.0) | 95 (7.2) | 54 (4.1) | 12 (0.9) | 11 (0.8) |
| Beauty products | 1415 | 6 (0.4) | 68 (4.8) | 152 (10.7) | 337 (23.8) | 291 (20.6) | 275 (19.4) | 156 (11.0) | 91 (6.4) | 19 (1.3) | 18 (1.3) | 2 (0.1) |
| Skincare products | 2315 | 2 (0.1) | 109 (4.7) | 150 (6.5) | 327 (14.1) | 578 (25.0) | 741 (32.0) | 197 (8.5) | 158 (6.8) | 34 (1.5) | 13 (0.6) | 6 (0.3) |
| Perfumes and colognes | 240 | 0 | 2 (0.8) | 2 (0.8) | 29 (12.1) | 6 (2.5) | 28 (11.7) | 15 (6.3) | 54 (22.5) | 38 (15.8) | 29 (12.1) | 37 (15.4) |
| Oral care products | 976 | 0 | 20  (2.0) | 42 (4.3) | 547 (56.0) | 300 (30.7) | 15 (1.5) | 52 (5.3) | 0 | 0 | 0 | 0 |
| Intimate care products | 7 | 0 | 0 | 1 (14.3) | 0 | 6 (85.7) | 0 | 0 | 0 | 0 | 0 | 0 |
| Other PCPs | 68 | 0 | 8 (11.8) | 6 (8.8) | 8  (11.8) | 21 (30.9) | 16 (23.5) | 2  (2.9) | 5 (7.4) | 2 (2.9) | 0 | 0 |

| **Supplemental Table 3: Use of personal care products (PCPs) with low (0-2), medium (3-6), and high (7-10) EWG Skin Deep® hazard scores in relation to race/ethnicity and product type.** | | | | | | | | |
| --- | --- | --- | --- | --- | --- | --- | --- | --- |
|  | **Skin Deep® Hazard score grouping** | **Products used in the last 24-48 hours by participant self-reported race and ethnicity, n (%)** | | | | | | |
|  |  | **All products used by participants**  **N=6334** | **Products used by Asian/Pacific Islander participants**  **n = 1138** | **Products used by Hispanic participants**  **n = 368** | **Products used by non-Hispanic Black participants**  **n=712** | **Products used by non-Hispanic White participants**  **n = 3504** | **Products used by Multiracial or other participants^1^,**  **n = 612** | ***P*^2^** |
| PCPs, overall | Low | 653 (10.3%) | 105 (9.2%) | 38 (10.3%) | 74 (10.4%) | 360 (10.3%) | 76 (12.4%) | <0.01 |
|  | Medium | 5003 (79.0%) | 907 (79.7%) | 292 (79.3%) | 531 (74.6%) | 2800 (79.9%) | 473 (77.3%) |  |
|  | High | 678 (10.7%) | 126 (11.1%) | 38 (10.3%) | 107 (15.0%) | 344 (9.8%) | 63 (10.3%) |  |
| Hair products | Low | 85 (6.5%) | 14 (6.4%) | 7 (9.2%) | 9 (6.2%) | 44 (5.9%) | 11 (8.2%) | <0.01 |
|  | Medium | 1057 (80.4%) | 181 (82.6%) | 64 (84.2%) | 96 (66.2%) | 606 (81.9%) | 110 (82.1%) |  |
|  | High | 172 (13.1%) | 24 (11.0%) | 5 (6.6%) | 40 (27.6%) | 90 (12.2%) | 13 (9.7%) |  |
| Beauty products^3^ | Low | 226 (16.0%) | 33 (13.8%) | 13 (15.3%) | 29 (20.3%) | 128 (15.5%) | 23 (19.5%) | 0.60 |
|  | Medium | 1058 (74.8%) | 183 (76.3%) | 64 (75.3%) | 103 (72.0%) | 625 (75.5%) | 83 (70.3%) |  |
|  | High | 130 (9.2%) | 24 (10.0%) | 8 (9.4%) | 11 (7.7%) | 75 (9.1%) | 12 (10.2%) |  |
| Skincare products^4^ | Low | 261 (11.3%) | 45 (10.5%) | 13 (9.8%) | 28 (10.6%) | 145 (11.5%) | 30 (13.3%) | 0.95^5^ |
|  | Medium | 1843 (79.6%) | 342 (79.9%) | 107 (80.5%) | 210 (79.5%) | 1011 (80.0%) | 173 (76.5%) |  |
|  | High | 211 (9.1%) | 41 (9.6%) | 13 (9.8%) | 26 (7.5%) | 108 (8.5%) | 23 (10.2%) |  |
| Perfumes and colognes | Low | 4 (1.7%) | 0 (0.0%) | 1 (5.9%) | 1 (2.7%) | 1 (0.9%) | 1 (4.0%) | 0.55 |
|  | Medium | 78 (32.5%) | 13 (27.1%) | 5 (29.4%) | 8 (21.6%) | 43 (38.1%) | 9 (36.0%) |  |
|  | High | 158 (65.8%) | 35 (72.9%) | 11 (64.7%) | 28 (75.7%) | 69 (61.1%) | 15 (60.0%) |  |
| Oral care products | Low | 62 (6.4%) | 11 (5.7%) | 3 (5.5%) | 6 (5.7%) | 34 (6.5%) | 8 (8.0%) | 0.36 |
|  | Medium | 914 (93.6%) | 183 (94.3%) | 52 (94.5%) | 99 (94.3%) | 488 (93.5%) | 92 (92.0%) |  |
|  | High | 0 (0.0%) | 0 (0.0%) | 0 (0.0%) | 0 (0.0%) | 0 (0.0%) | 0 (0.0%) |  |
| Intimate care products^5^ | Low | 1 (14.3%) | 1 (50.0%) | 0 (0.0%) | 0 (0%) | 0 (0.0%) | 0 (0.0%) | 0.17^5^ |
|  | Medium | 6 (85.7%) | 1 (50.0%) | 0 (0.0%) | 2 (100.0%) | 1 (100.0%) | 2 (100.0%) |  |
|  | High | 0 (0.0%) | 0 (0.0%) | 0 (0.0%) | 0 (0.0%) | 0 (0.0%) | 0 (0.0%) |  |
| Other PCPs | Low | 14 (20.6%) | 1 (14.3%) | 1 (50.0%) | 1 (6.3%) | 8 (22.2%) | 3 (42.9%) | <0.01 |
|  | Medium | 47 (69.1%) | 4 (57.1%) | 0 (0.0%) | 13 (81.3%) | 26 (72.2%) | 4 (57.1%) |  |
|  | High | 7 (10.3%) | 2 (28.6%) | 1 (50.0%) | 2 (12.5%) | 2 (5.6%) | 0 (0.0%) |  |
| 1. Multiracial or other groups category included those reporting more than one race, American Indian or Alaska Native, and prefer not to answer or no response 2. P-values based on chi-squared tests comparing percentage of products within a given hazard category across race/ethnicities unless otherwise noted. 3. Beauty products included face makeup, eye makeup, lip products, and nail products. 4. Skincare products included rinse-off and leave-on facial skin care products, rinse-off body cleansing products, skin moisturizing products, at-home hair removal products, antiperspirants and/or deodorants, and sunscreen, sun tanning, after sun products and/or sunless tanning products. 5. P-value based on Fisher’s exact test. 6. Intimate care products include intimate wash and wipe products. | | | | | | | | |

| **Supplemental Table 4. Distribution of EWG Skin Deep® hazard scores (low, moderate, and high) of personal care products (PCPs) used within the last 24-48 hours in relation to participant sociodemographic characteristics.^1,2^** | | | | |
| --- | --- | --- | --- | --- |
|  | **PCPs, all (n=6334)** | **Low hazard (0-2)**  **(n=653)** | **Moderate hazard**  **(3-6)**  **(n=5003)** | **High hazard**  **(7-10)**  **(n=678)** |
|  | N | N (row %) | N (row %) | N (row %) |
| Age (years) |  |  |  |  |
| 18-39 | 3794 | 412 (10.9%) | 2974 (78.4%) | 408 (10.8%) |
| 40-59 | 1757 | 169 (9.6%) | 1391 (79.2%) | 197 (11.2%) |
| ≥60 | 778 | 72 (9.3%) | 634 (81.5%) | 72 (9.3%) |
| p-value | **<0.01** |  |  |  |
| Education |  |  |  |  |
| Less than bachelors | 1635 | 146 (8.9%) | 1314 (80.4%) | 175 (10.7%) |
| Bachelors | 2123 | 222 (10.5%) | 1667 (78.5%) | 234 (11%) |
| More than bachelors | 2558 | 279 (10.9%) | 2012 (78.7%) | 267 (10.4%) |
| p-value | *0.06* |  |  |  |
| Race and ethnicity |  |  |  |  |
| AAPI | 1138 | 105 (9.2%) | 907 (79.7%) | 126 (11.1%) |
| Hispanic | 368 | 38 (10.3%) | 292 (79.3%) | 38 (10.3%) |
| NHB | 712 | 74 (10.4%) | 531 (74.5%) | 107 (15.1%) |
| NHW | 3504 | 360 (10.3%) | 344 (78.9%) | 344 (10.8%) |
| Multiracial or other^3^ | 612 | 76 (12.4%) | 63 (76.7%) | 63 (10.3%) |
| p-value | **0.01** |  |  |  |
| Gender identity |  |  |  |  |
| Female | 5805 | 608 (10.5%) | 4558 (78.5%) | 639 (11.0%) |
| Male | 529 | 45 (8.5%) | 446 (84.3%) | 39 (7.4%) |
| p-value | **<0.01** |  |  |  |
| Natality |  |  |  |  |
| US-born | 5262 | 551 (10.5%) | 4165 (79.2%) | 546 (10.4%) |
| Foreign-born | 1011 | 97 (9.6%) | 791 (78.2%) | 123 (12.2%) |
| p-value | 0.28 |  |  |  |
| Marital status |  |  |  |  |
| Married | 2398 | 248 (10.3%) | 1916 (79.9%) | 234 (9.8%) |
| Non-Married^4^ | 3936 | 405 (10.3%) | 3087 (78.5%) | 444 (11.3%) |
| p-value | 0.19 |  |  |  |
| Household income ($) |  |  |  |  |
| <$50,000 | 890 | 108 (12.1%) | 676 (76%) | 106 (11.9%) |
| $50,000 - $74,999 | 981 | 94 (9.6%) | 761 (77.6%) | 126 (12.8%) |
| $75,000 - $99,999 | 989 | 110 (11.1%) | 776 (78.5%) | 103 (10.4%) |
| ≥$100,000 | 3397 | 329 (9.7%) | 2731 (80.4%) | 337 (9.9%) |
| p-value | 0.11 |  |  |  |
| Role at university |  |  |  |  |
| Undergraduate | 892 | 90 (10.1%) | 719 (80.6%) | 83 (9.3%) |
| Graduate student | 1459 | 149 (10.2%) | 1144 (78.4%) | 166 (11.4%) |
| Faculty | 893 | 93 (10.4%) | 723 (80.9%) | 77 (8.6%) |
| Staff | 2858 | 299 (10.5%) | 2227 (77.9%) | 332 (11.6%) |
| Other/unknown | 232 | 22 (9.5%) | 190 (81.9%) | 20 (8.6%) |
| p-value | 0.40 |  |  |  |
| Abbreviations: AAPI, Asian American/Pacific Islander; NHB, non-Hispanic Black; NHW, non-Hispanic White   1. Some rows may not add up to exactly 100% due to missing data. 2. P-values are based on Kruskal-Wallis tests. Associations significant at P<0.05 are bolded and those at P<0.10 are italicized. 3. Multiracial or other race category included those reporting more than one race, American Indian or Alaska Native, and prefer not to answer or no response. 4. Non-married category included those who reported being single/never married, widowed, separated, or divorced. | | | | |

| **Supplemental Table 5: Multivariable logistic regression models examining relative risk (RR) of using at least one personal care product (PCP) with a high (7-10) EWG Skin Deep score within the last 24-48 hours in relation to sociodemographic characteristics (N=593).^1^** | | | | | |
| --- | --- | --- | --- | --- | --- |
|  | **All products** | **Hair products** | **Beauty products** | **Skin care products** | **Perfumes/colognes** |
|  | **RR (95% CI)** | **RR (95% CI)** | **RR (95% CI)** | **RR (95% CI)** | **RR (95% CI)** |
| **Gender** |  |  |  |  |  |
| Female | REF | REF | REF | REF | REF |
| Male | **0.65 (0.49, 0.86)** | **0.51 (0.26, 0.99)** | 0.69 (0.28, 1.66) | *0.65 (0.40, 1.04)* | 0.82 (0.56, 1.20) |
| **Age (years)** |  |  |  |  |  |
| 18-39 | REF | REF | REF | REF | REF |
| 40-59 | 1.08 (0.93, 1.26) | **1.55 (1.09, 2.19)** | 1.19 (0.79, 1.79) | 0.89 (0.63, 1.27) | 0.87 (0.70, 1.08) |
| 60+ | 0.96 (0.76, 1.21) | 1.40 (0.86, 2.29) | 0.93 (0.50, 1.71) | 1.35 (0.92, 1.98) | **0.55 (0.32, 0.95)** |
| **Race and ethnicity** |  |  |  |  |  |
| NHW | REF | REF | REF | REF | REF |
| AAPI | 0.83 (0.77, 1.13) | 1.04 (0.67, 1.63) | 0.94 (0.60, 1.48) | 0.80 (0.55, 1.18) | 1.02 (0.87, 1.20)^4^ |
| Hispanic | 0.87 (0.68, 1.13) | 0.77 (0.41, 1.45) | 0.84 (0.46, 1.52) | 0.88 (0.55, 1.40) |  |
| NHB | **1.23 (1.03, 1.46)** | **1.99 (1.37, 2.89)** | 0.67 (0.34, 1.33) | 1.30 (0.89, 1.90) |  |
| Multiracial/other^2^ | 1.14 (0.88, 1.46) | 0.97 (0.46, 2.04) | 1.23 (0.64, 2.36) | 1.20 (0.74, 1.95) |  |
| **Marital status** |  |  |  |  |  |
| Non-married^3^ | REF | REF | REF | REF | REF |
| Married | 0.92 (0.79, 1.08) | 1.14 (0.81, 1.61) | 0.79 (0.53, 1.19) | *0.71 (0.51, 1.00)* | 0.92 (0.75, 1.12) |
| **Household income** |  |  |  |  |  |
| <$50,000 | 0.95 (0.78, 1.16) | 1.10 (0.69, 1.76) | 0.83 (0.48, 1.43) | 1.09 (0.75, 1.58) | 0.99 (0.79, 1.24) |
| $50,000 - $74,999 | 1.05 (0.87, 1.26) | 1.08 (0.69, 1.68) | 0.90 (0.55, 1.48) | *1.34 (0.95, 1.88)* | 1.04 (0.85, 1.27) |
| $75,000 - $99,999 | 1.02 (0.85, 1.22) | 1.28 (0.87, 1.89) | 0.77 (0.46, 1.26) | 0.92 (0.62, 1.38) | 0.98 (0.77, 1.25) |
| $100,000+ | REF | REF | REF | REF | REF |
| Abbreviations: AAPI, Asian American/Pacific Islander; NHB, non-Hispanic Black; NHW, non-Hispanic White   1. Models are mutually adjusted for all sociodemographic factors in the table (e.g., gender, age, race/ethnicity, marital status, income). Associations significant at p<0.05 are bolded and those with p<0.10 are italicized. 2. Multiracial or other race and ethnicity category included those reporting more than one race or multiracial (52 [7.9%]), American Indian or Alaska Native (2 [3.1%]), and unknown (those who provided no response to race and/or ethnicity or responded, ‘prefer not to answer’ (11 [16.9%]). 3. Non-married category included those who reported being single/never married, widowed, separated, or divorced. 4. Due to issues around non-convergence, race categories were collapsed for this model. | | | | | |

| **Supplemental Table 6: Multivariable logistic regression models examining relative risk (RR) of using at least one personal care product (PCP) with a high EWG Skin Deep® hazard score (7-10) in relation to participants' perceptions and attitudes about PCP safety and regulation (n=581).** | |
| --- | --- |
| **Perceptions** | **Relative Risk (95%CI)^1^** |
| The personal care products I use affect my health.  Strongly disagree  Disagree  Neither agree nor disagree  Agree  Strongly agree | 0.88 (0.64, 1.20)  1.03 (0.87, 1.23)  REF  0.93 (0.80, 1.08)  0.83 (0.67, 1.04) |
| Organic, natural, non-toxic, or eco-friendly personal care products have fewer toxic chemicals than regular products.  Strongly disagree  Disagree  Neither agree nor disagree  Agree  Strongly agree | 0.51 (0.23, 1.15)  1.08 (0.87, 1.33)  REF  0.95 (0.82, 1.10)  0.84 (0.67, 1.04) |
| Consumers should be concerned about the health effects of personal care products.^2^  Disagree/Strongly disagree  Neither agree nor disagree  Agree  Strongly agree | *0.59 (0.32, 1.10)*  REF  0.91 (0.77, 1.08)  **0.80 (0.65, 0.98)** |
| There is no reason to worry about the health effects from chemicals that might be in personal care products.^3^  Strongly disagree  Disagree  Neither agree nor disagree  Agree/Strongly agree | 0.89 (0.70, 1.12)  1.00 (0.81, 1.24)  REF  1.11 (0.79, 1.54) |
| Overall, the benefits of using personal care products outweigh any risks from exposure to toxic chemicals that might be in these products.  Strongly disagree  Disagree  Neither agree nor disagree  Agree  Strongly agree | 0.85 (0.68, 1.07)  1.06 (0.92, 1.23)  REF  0.91 (0.72, 1.14)  1.19 (0.73, 1.94) |
| Organic, natural, non-toxic, or eco-friendly personal care products are just as effective as regular products.^4^  Disagree/Strongly disagree  Neither agree nor disagree  Agree/Strongly agree | 0.92 (0.78, 1.07)  REF  **0.79 (0.68, 0.90)** |
| The Food and Drug Administration (FDA) and other government agencies do a good job of regulating personal care products to ensure they are safe for consumers.  Strongly disagree  Disagree  Neither agree nor disagree  Agree  Strongly agree | 0.94 (0.77, 1.16)  1.00 (0.86, 1.18)  REF  1.03 (0.85, 1.24)  1.10 (0.69, 1.75) |
| 1 Models are adjusted for gender, age, race/ethnicity, marital status, and income. Associations significant at p<0.05 are bolded and those with p<0.10 are italicized.  2 For this model, disagree and strongly disagree responses were collapsed due to data sparseness.  3 For this model, agree and strongly agree responses were collapsed due to data sparseness.  4 For this model, strongly disagree/disagree and strongly agree/agree responses were collapsed due to data sparseness. | |

| **Supplemental Table 7: Adjusted linear regression models examining the relationship between the mean hazard scores of hair products, skin care products, and beauty products used by participants in relation to self-reported personal care product (PCP) purchasing behaviors^1,2^** | | | | | | |
| --- | --- | --- | --- | --- | --- | --- |
|  | **Hair products** | |  | **Skin products** |  | **Beauty products** |
|  | **n** | **β (95% CI)** | **n** | **β (95% CI)** | **n** | **β (95% CI)** |
| **Use a healthy product app or website** | 519 |  | 538 |  | 418 |  |
| Always/usually |  | *-0.30 (-1.10, 0.68)* |  | **-0.49 (-0.77, -0.21)** |  | -0.31 (-0.75, 0.12) |
| Sometimes |  | *0.35 (-0.38, 1.96)* |  | -0.02 (-0.29, 0.24) |  | *-0.35 (-0.76, 0.05)* |
| Rarely/never |  | REF |  | REF |  | REF |
| **Read the ingredients on product labels** | 527 |  | 550 |  | 423 |  |
| Always/usually |  | **-0.27 (-0.49, -0.04)** |  | **-0.26 (-0.82, -0.30)** |  | **-0.40 (-0.67, -0.14)** |
| Sometimes |  | *-0.24 (-0.50 0.03)* |  | -0.13 (-0.33, 0.07) |  | *-0.26 (-0.56, 0.04)* |
| Rarely/never |  | REF |  | REF |  | REF |
| **Seek products with labels indicating**  **natural, non-toxic, or eco-friendly**  **ingredients** | 527 |  | 548 |  | 423 |  |
| Always/usually |  | -0.17 (-0.39, 0.06) |  | **-0.17 (-0.36, -0.01)** |  | **-0.34 (-0.62, -0.06)** |
| Sometimes |  | -0.06 (-0.32, 0.20) |  | 0.05 (-0.15, 0.25) |  | -0.13 (-0.42, 0.16) |
| Rarely/never |  | REF |  | REF |  | REF |

1. Models adjusted for age, race/ethnicity, marital status, gender and income.
2. Associations significant at P<0.05 are bolded and those with P<0.10 are italicized.

| **Supplemental Table 8: Adjusted logistic regression models examining relative risk (RR) of using at least one product with a high EWG Skin Deep® hazard score (7-10) in relation to self-reported personal care product (PCP) purchasing behaviors^1,2^ .** | | | | | | |
| --- | --- | --- | --- | --- | --- | --- |
|  | **Hair products** | |  | **Skin products** |  | **Beauty products** |
|  | **n** | **RR (95% CI)** | **n** | **RR (95% CI)** | **n** | **RR (95% CI)** |
| **Use a healthy product app or website** | 519 |  | 538 |  | 418 |  |
| Always/usually |  | 0.82 (0.45,1.48) |  | 0.78 (0.45, 1.36) |  | 1.02 (0.54, 1.92) |
| Sometimes |  | 1.05 (0.62, 1.80) |  | 0.88 (0.54, 1.44) |  | 1.03 (0.58, 1.83) |
| Rarely/never |  | REF |  | REF |  | REF |
| **Read the ingredients on product labels** | 527 |  | 550 |  | 423 |  |
| Always/usually |  | 0.84 (0.60, 1.17) |  | 0.88 (0.65, 1.19) |  | 0.86 (0.58, 1.29) |
| Sometimes |  | **0.66 (0.42, 1.04)** |  | 0.98 (0.70, 1.37) |  | 1.1 (0.73, 1.66) |
| Rarely/never |  | REF |  | REF |  | REF |
| **Seek products with labels indicating**  **natural, non-toxic, or eco-friendly**  **ingredients** | 527 |  | 548 |  | 423 |  |
| Always/usually |  | 0.95 (0.68, 1.34) |  | 1.12 (0.80, 1.55) |  | 0.87 (0.56, 1.34) |
| Sometimes |  | 0.96 (0.64, 1.42) |  | **1.39 (1.00, 1.94)** |  | 1.35 (0.92, 1.99) |
| Rarely/never |  | REF |  | REF |  | REF |

1. Models adjusted for age, race/ethnicity, marital status, gender and income.
2. Associations significant at P<0.05 are bolded and those with P<0.10 are italicized.

| **Supplemental Table 9: Logistic regression models examining risk of using at least one personal care product (PCP) with a high EWG Skin Deep hazard score (7-10) in relation to participants' perceptions and attitudes about PCP safety and regulation (n=581).** | |
| --- | --- |
| **Perceptions** | **Relative Risk (95%CI)^1^** |
| The personal care products I use affect my health.  Strongly disagree  Disagree  Neither agree nor disagree  Agree  Strongly agree | 0.88 (0.64, 1.20)  1.03 (0.87, 1.23)  REF  0.93 (0.80, 1.08)  0.83 (0.67, 1.04) |
| Organic, natural, non-toxic, or eco-friendly personal care products have fewer toxic chemicals than regular products.  Strongly disagree  Disagree  Neither agree nor disagree  Agree  Strongly agree | 0.51 (0.23, 1.15)  1.08 (0.87, 1.33)  REF  0.95 (0.82, 1.10)  0.84 (0.67, 1.04) |
| Consumers should be concerned about the health effects of personal care products.^2^  Disagree/Strongly disagree  Neither agree nor disagree  Agree  Strongly agree | *0.59 (0.32, 1.10)*  REF  0.91 (0.77, 1.08)  **0.80 (0.65, 0.98)** |
| There is no reason to worry about the health effects from chemicals that might be in personal care products.^3^  Strongly disagree  Disagree  Neither agree nor disagree  Agree/Strongly agree | 0.89(0.70, 1.12)  1.00 (0.81, 1.24)  REF  1.11 (0.79, 1.54) |
| Overall, the benefits of using personal care products outweigh any risks from exposure to toxic chemicals that might be in these products.  Strongly disagree  Disagree  Neither agree nor disagree  Agree  Strongly agree | 0.85 (0.68, 1.07)  1.06 (0.92, 1.23)  REF  0.91 (0.72, 1.14)  1.19 (0.73, 1.94) |
| Organic, natural, non-toxic, or eco-friendly personal care products are just as effective as regular products.^4^  Disagree/Strongly disagree  Neither agree nor disagree  Agree/Strongly agree | 0.92 (0.78, 1.07)  REF  **0.79 (0.68, 0.90)** |
| The Food and Drug Administration (FDA) and other government agencies do a good job of regulating personal care products to ensure they are safe for consumers.  Strongly disagree  Disagree  Neither agree nor disagree  Agree  Strongly agree | 0.94 (0.77, 1.16)  1.00 (0.86, 1.18)  REF  1.03 (0.85, 1.24)  1.10 (0.69, 1.75) |

1. Models are adjusted for gender, age, race/ethnicity, marital status, and income. Associations significant at p<0.05 are bolded and those with p<0.10 are italicized.

2 For this model, disagree and strongly disagree responses were collapsed due to data sparseness.

3 For this model, agree and strongly agree responses were collapsed due to data sparseness.

4 For this model, strongly disagree/disagree and strongly agree/agree responses were collapsed due to data sparseness.
